# Effects of Injectable Solutions on the Quality of Monocyte-Derived Dendritic Cells for Immunotherapy

**DOI:** 10.1155/2024/6817965

**Published:** 2024-06-07

**Authors:** Laís Teodoro Da Silva, Bruna Tiaki Tiyo, Silvia de Jesus Mota, Marina Mazzilli Ortega, Gabriela Justamante Handel Schmitz, Noemi Nosomi Taniwaki, Gislene Mitsue Namiyama Nishina, Alberto José da Silva Duarte, Telma Miyuki Oshiro

**Affiliations:** ^1^ Laboratory of Medical Investigation in Dermatology and Immunodeficiencies (LIM-56) Clinical Hospital HCFMUSP Faculty of Medicine University of Sao Paulo, Sao Paulo, SP, Brazil; ^2^ Electron Microscopy Nucleus Adolfo Lutz Institute, Sao Paulo, Brazil

## Abstract

Therapeutic vaccines based on monocyte-derived dendritic cells have been shown to be promising strategies and may act as complementary treatments for viral infections, cancers, and, more recently, autoimmune diseases. Alpha-type-1-polarized dendritic cells (aDC1s) have been shown to induce type-1 immunity with a high capacity to produce interleukin-12p70 (IL-12p70). In the clinical use of cell-based therapeutics, injectable solutions can affect the morphology, immunophenotypic profile, and viability of cells before delivery and their survival after injection. In this sense, preparing a cell suspension that maintains the quality of aDC1s is essential to ensure effective immunotherapy. In the present study, monocytes were differentiated into aDC1s in the presence of IL-4 and GM-CSF. On day 5, the cells were matured by the addition of a cytokine cocktail consisting of IFN-*α*, IFN-*γ*, IL-1*β*, TNF-*α*, and Poly I:C. After 48 hr, mature aDC1s were harvested and suspended in two different solutions: normal saline and Ringer's lactate. The maintenance of cells in suspension was evaluated after 4, 6, and 8 hr of storage. Cell viability, immunophenotyping, and apoptosis analyses were performed by flow cytometry. Cellular morphology was observed by electron microscopy, and the production of IL-12p70 by aDC1s was evaluated by ELISA. Compared with normal saline, Ringer's lactate solution was more effective at maintaining DC viability for up to 8 hr of incubation at 4 or 22°C.

## 1. Introduction

Dendritic cells (DCs) are multifunctional cells capable of priming and maintaining a specific immune response [1]. Based on their subtypes and sense of environmental signals, DCs can interact with naïve T cells and produce various immune mediators that are responsible for activating cellular responses or inducing immune tolerance. As such, within the past 20 years, DC-based immunotherapy has become an interesting approach in the context of viral infections and cancers due to its ability to activate T-cell responses, and more recently, DC populations with immunosuppressive activities have been studied for clinical applications in patients with autoimmune conditions.

For immunotherapeutic protocols in which many cells are needed, due to their high level of plasticity, DCs can be generated *ex vivo* from precursors such as hematopoietic progenitor cells or monocytes using different cytokine combinations. In this context, alpha-type-1 polarized dendritic cells (aDC1s) have been studied for their clinically relevant characteristics and show a high capacity to produce interleukin-12p70 (IL-12p70) and, therefore, induce type-1 T-cell immunity, which is effective for fighting cancers and viral infections [2].

Variable clinical outcomes have been observed in clinical trials using DCs. This variability can be attributed to several factors, such as the type of disease/patient status, protocol for obtaining/activating DCs, immunization route, and number of cells/doses used. In addition, the quality of DCs, such as viability level and activation status, is crucial for good performance and can interfere with clinical outcomes.

Quality control for DC viability and evaluation of activation status, as well as product sterility testing, are mandatory in the clinical adoption of cell-based therapeutics for releasing vaccines. These tests include tests for the viability and maturation status of DCs and the absence of endotoxin, mycoplasma, and bacterial/fungal contamination, and these tests must be performed on the final product. Completion of these tests can take several hours, during which time the formulated vaccine must be kept in adequate condition until it is infused into the patient.

Because cell cultivation medium cannot be inoculated into patients, it should be replaced with a suitable solution for injection before the quality control test. This solution must be able to maintain cell integrity during the time necessary for the completion of the tests until inoculation.

In the literature, little information can be found about the influence of temperature and injectable solutions on the quality of cell-based immunotherapy. Normal saline is often used as a vehicle for the injection of therapeutic drugs, including immunotherapy using DCs, with [3, 4, 5, 6, 7, 8, 9, 10, 11, 12] or without [13, 14, 15, 16, 17, 18, 19, 20, 21, 22, 23] the addition of human ALB. In turn, Ringer's lactate solution is a type of isotonic, crystalloid fluid that presents a balanced pH and is clinically used for fluid replacement, similar to normal saline [24].

In this sense, it is of fundamental importance to define both the injectable solution and the storage temperature to guarantee the stability and quality of the cell therapy product. The present study describes the preservation of aDC1s in Ringer's lactate compared to that in normal saline at different temperatures through analysis of cell viability, immunophenotyping, apoptosis, electron microscopy, and IL-12p70 production.

## 2. Materials and Methods

### 2.1. Sample Collection

Samples obtained from 19 healthy volunteers were included in this study. The local ethics committee (CAPPesq 6.020.101) approved this study.

By centrifugation through a Ficoll-Paque Plus (Cytiva) gradient, peripheral blood mononuclear cells (PBMCs) were isolated from the leukoreduction system chambers and recovered after routine donor plateletpheresis, followed by storage in liquid nitrogen until use.

### 2.2. aDC1 Generation

Monocytes obtained by plastic adherence [25] were differentiated into aDC1s as described previously [2]. Briefly, monocytes were cultured in AIM-V serum-free medium (Thermo Fisher Scientific) supplemented with 100 ng/ml recombinant human GM-CSF and 200 ng/ml recombinant human IL-4 (PeproTech) for 5 days to obtain immature aDC1s. After incubation, the cells were stimulated using a proinflammatory cocktail composed of 1,000 UI/ml IFN-*α* 2b (Miltenyi Biotec), 1,000 UI/ml IFN-*γ* (PeproTech), 20 *µ*g/ml Poly I:C (Sigma), 10 ng/ml IL-1*β* (PeproTech), and 25 ng/ml TNF-*α* (Peprotech). After 48 hr, mature aDC1s were harvested, washed three times with normal saline, and evaluated after 4, 6, and 8 hr of storage in a syringe or 24-well ultralow attachment plates in two different injectable solutions, namely, normal saline and Ringer's lactate (both obtained from Baxter).

### 2.3. aDC1 Immunophenotypic Characterization

Immature and mature aDC1s were stained with Fixable Aqua Dead Cell Stain (Thermo Fisher) to determine cell viability and for the surface markers CD14 PE (clone M5E2), CD11c PE CF594 (clone B-ly6), HLA-DR Alexa Fluor 700 (clone G46–6), CCR7 V450 (clone 150503), CD86 PE-Cy7 (clone 2331), CD83 APC (clone HB15e), and CD40 FITC (clone 5C3) (BD Biosciences) before and after storage. The analysis strategy applied to define the aDC1 surface molecules is depicted in *Supplementary figure 1*. aDC1s were acquired on an LSR Fortessa (BD Bioscience) using DIVA software, and the analysis was performed with FlowJo vX 0.7 software (TreeStar, OR, USA) and GraphPad Prism v.8 software (GraphPad Software Inc., CA, USA).

### 2.4. Analysis of Apoptosis and Late Apoptosis/Necrosis in aDC1 Cells

aDC1s were stained with Annexin V-FITC and 7-AAD (BD Biosciences) to detect early- (7-AAD negative and Annexin V positive) and late-stage cell apoptosis (7-AAD and Annexin V double positive) by flow cytometry according to the manufacturer's instructions. Briefly, the cells were washed twice with cold PBS and then resuspended in 1x binding buffer (0.1 M HEPES (pH 7.4), 1.4 M NaCl, and 25 mM CaCl_2_). Thereafter, Annexin V and 7-AAD were added, and aDC1 cells were incubated for 15 min at room temperature. After incubation, the cells were immediately acquired on an LSR Fortessa flow cytometer.

### 2.5. Transmission Electron Microscopy Analysis

aDC1s were stored in 24-well ultralow plates for 4, 6, and 8 hr in normal saline or Ringer's lactate solutions at 4, 22, and 37°C. The pellets were washed, fixed in 2.5% glutaraldehyde in 0.1 M sodium cacodylate buffer (pH 7.2), rinsed in the same buffer, postfixed in 1% osmium tetroxide, dehydrated in an acetone series, and embedded in Epon resin. Ultrathin sections were obtained with a Sorvall ultramicrotome, stained with uranyl acetate and lead citrate, and observed under a JEOL transmission electron microscope operating at 80 kV. Images were recorded with a Gatan 785 ES1000W Erlangshen camera. Unstored cells were used as controls.

### 2.6. Interleukin-12p70 Production by aDC1

After 4, 6, and 8 hr of storage with normal saline or Ringer's lactate, 2.5 × 10^4^ aDC1 cells obtained from the seventh day of culture were incubated in a 96-well plate for 24 hr with 250 ng/ml rCD40L (ALX-522-110, Enzo Life Sciences, NY, USA) to mimic the interaction between aDC1 and Th cells expressing CD40L. After incubation, the supernatants were harvested and stored at −80°C for analysis of IL-12p70 in triplicate by ELISA (Human IL-12p70 ELISA Ready-SET Go! Kit®, eBioscience, San Diego, CA, USA) according to the manufacturer's instructions.

### 2.7. Statistical Analysis

Statistical analysis was performed using GraphPad Prism v.8. Data are represented by median values with interquartile ranges. The results were analyzed by one-way ANOVA and the nonparametric Kruskal‒Wallis test. *p*  < 0.05 was considered to indicate statistical significance.

## 3. Results

### 3.1. Normal Saline and Storage Temperature Can Impair aDC1 Viability

The vaccine product is obtained through aDC1 cultivation for 7 days in serum-free medium, which should be replaced with an injectable solution before being inoculated into the patient. In this context, viability and immunophenotyping were monitored after 4, 6, and 8 hr of aDC1 storage in ultralow attachment plates at 4, 22, and 37°C with normal saline or Ringer's lactate and compared with aDC1 before incubation (unstored cells).

**Figure 1:**
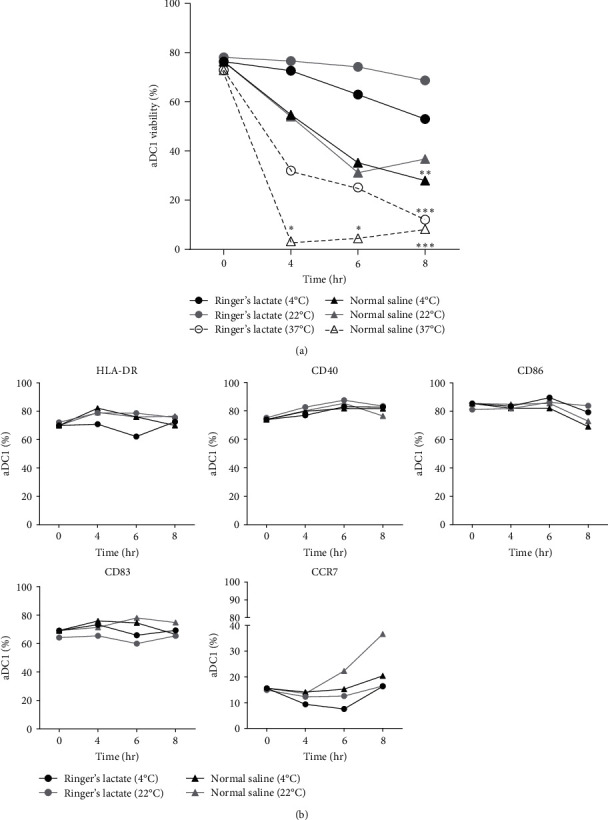
Effect of temperature and injectable solutions on aDC1 characteristics. aDC1s were verified before and after incubation at 4, 22, and 37°C with normal saline and Ringer's lactate. The average percentage of cell viability (a) and surface molecule expression by aDC1 (b) are represented by line graphs before (T 0 hr) and after 4, 6, and 8 hr of incubation with Ringer's lactate at 4°C (closed circles), 22°C (gray circles) and 37°C (open circles) or with normal saline at 4°C (closed triangles), 22°C (gray triangles) and 37°C (open triangles) (*n* = 19). One-way ANOVA and the nonparametric Kruskal‒Wallis test were used to calculate *p* values.  ^*∗*^*p*  < 0.05;  ^*∗∗*^*p*  < 0.005;  ^*∗∗∗*^*p*  < 0.001.

The viability of aDC1 before incubation was 78%. As shown in Figure 1(a), after 8 hr of incubation with Ringer's lactate at 37°C, the cell viability significantly decreased to 12% (*p*  < 0.001). Additionally, compared to that of unstored cells, the viability of aDC1s in normal saline at 4°C for 8 hr was reduced to 28% (*p*  < 0.005), and when aDC1s were preserved at 37°C with the same storage suspension vehicle, a significant decrease in viability was observed after 4 hr of incubation (*p*  < 0.05).

The induction of adequate T-cell responses requires viable DCs, as well as the upregulation of costimulatory molecules [26]. Based on these findings, an immunophenotypic analysis of aDC1s was performed, and the results are depicted in Figure 1(b). The results showed that the expression of surface molecules related to activation markers (HLA-DR, CD40, CD86, and CD83) and a cell migration marker (CCR7) was similar between the samples evaluated, independent of the injectable solution, showing no change in the percentage of cells expressing the studied markers compared to the percentage of unstored cells.

It is important to point out that, due to the viability being lower than 40%, it was not possible to analyze the immunophenotypic profile of aDC1s when kept at 37°C.

In general, whereas Ringer's lactate, used at both 4 and 22°C, seems to be suitable for maintaining aDC1 viability, a drastic reduction in cell viability was observed when aDC1 was maintained at 37°C in any of the injection solutions studied, accompanied by changes in its size, as observed in the flow cytometer dot plot parameters, mainly in relation to aDC1 forward scatter (FSC), characterized by a shift to the left of the studied population (*Supplementary figure 2*). These signals are suggestive of the early stages of apoptosis and will be studied in the following section.

### 3.2. Normal Saline Induces Late Apoptotic/Necrotic aDC1

**Figure 2:**
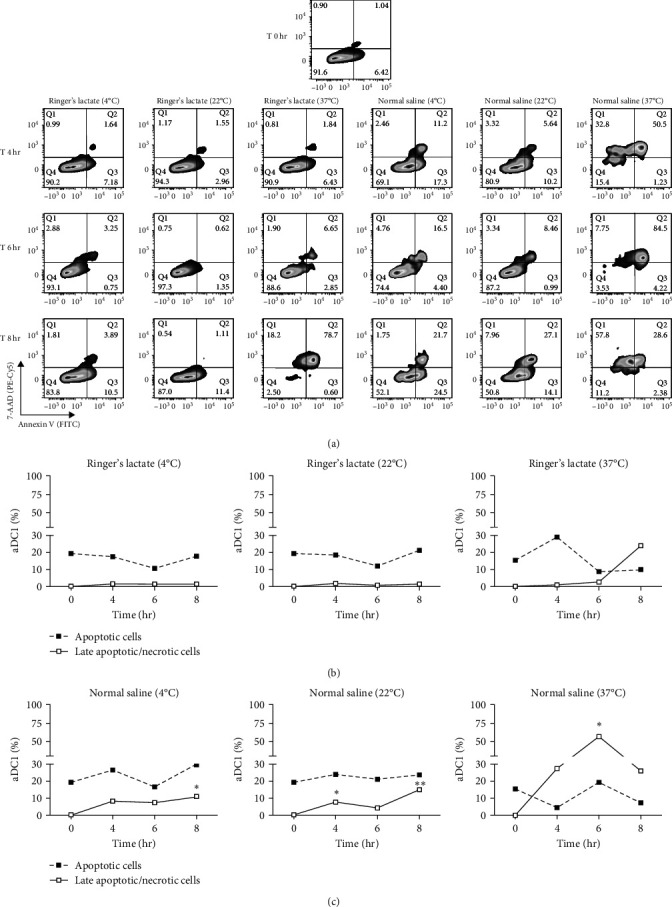
Analysis of apoptotic and late apoptotic/necrotic aDC1s after storage in injectable solutions. In (a), flow cytometry plots from one individual represented within the aDC1 population (SSC versus CD14 PE negative and SSC versus CD11c positive), live cells (7-AAD and Annexin V double negative), apoptotic (7-AAD negative and Annexin V positive) and late apoptotic/necrotic cells (7-AAD and Annexin V double positive) before (T 0 hr) and after 4 (T 4 hr), 6 (T 6 hr), and 8 hr (T 8 hr) of incubation with normal saline or Ringer's lactate at 4, 22, and 37°C. In (b, c), line graphs represent the median percentages of early apoptotic (closed squares) and late apoptotic/necrotic (open squares) cells before (0 hr) and after 4, 6, and 8 hr of incubation with Ringer's lactate (b) or with normal saline (c) at 4, 22, and 37°C, respectively (*n* = 7). One-way ANOVA and the nonparametric Kruskal‒Wallis test were used to calculate *p* values.  ^*∗*^*p*  < 0.05;  ^*∗∗*^*p*  < 0.005.

Death pathways based on caspase activation by phosphatidylserine (PS) exposure by Annexin V (early-stage cellular apoptosis) and plasma membrane permeabilization by 7-amino-actinomycin (7AA-D) (late-stage cellular apoptosis) were evaluated in aDC1s maintained in suspensions containing Ringer's lactate or normal saline at 4, 22, and 37°C in ultralow attachment plates. The maintenance of cells in these suspensions was evaluated after storage for 4, 6, and 8 hr and compared to that of unstored aDC1 (T 0 hr) (Figure 2).

Figure 2(a) shows flow cytometric dot plots representative of samples from one individual. Compared to the initial profile (T 0 hr), double-positive cells for Annexin V and 7-AAD, which are representative of late apoptotic/necrotic cells, increased over time for cultures incubated with saline at 4 and 22°C and, largely, at 37°C (*p*  < 0.05). The results for the cells incubated with Ringer's lactate were similar to those for the initial conditions at all the temperatures studied (Figure 2(b)).

The median percentage of aDC1s in late apoptotic/necrotic cells before replacement with the injectable solutions was 0.2%. After 8 hr of incubation with normal saline at 4 and 22°C, the percentages of aDC1s in the late stages of apoptosis significantly increased to 11% and 14.8%, respectively (*p*  < 0.05). Furthermore, when preserved at 37°C with the same suspension vehicle for 6 hr, 25.9% of the aDC1s exhibited late apoptosis/necrosis (*p*  < 0.05) (Figure 2(c)).

Unlike Ringer's lactate, normal saline induced late apoptosis/necrosis in aDC1 cells maintained at the different temperatures studied.

### 3.3. Both Solutions Induce a Progressive Increase in aDC1 Deterioration over Time

**Figure 3:**
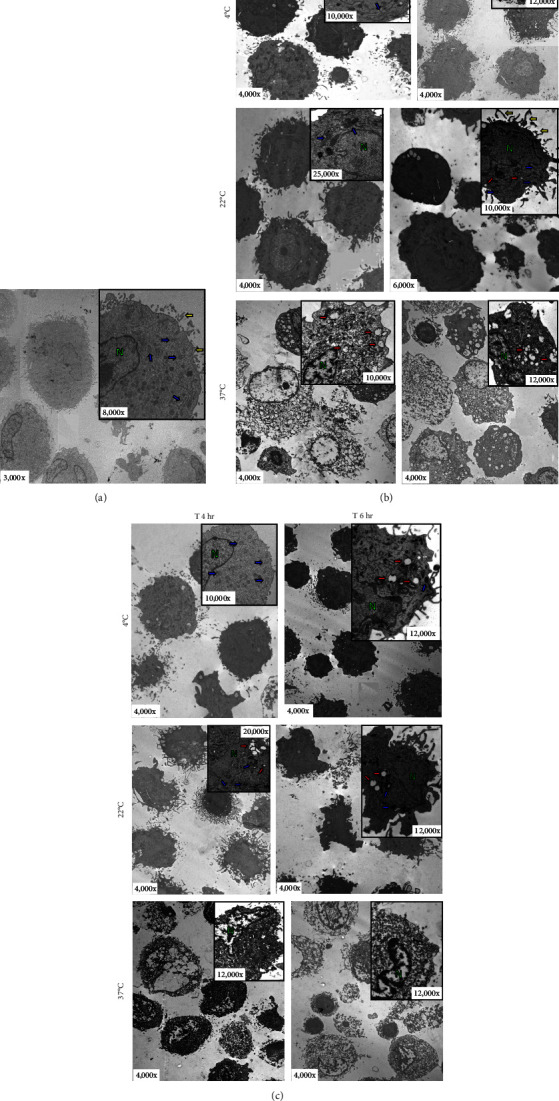
Transmission electron microscopy images. The aDC1 images are representative of one individual (unstored cells) before (a) and after incubation at 4, 22, and 37°C with Ringer's lactate (b) or normal saline (c) during 4 and 6 hr. Cell nucleus (N); blue arrows point to organelles (mitochondria and endoplasmic reticulum); yellow and red arrows point to dendrites and vacuoles or vesicles, respectively.

Transmission electron microscopy (TEM) was used to analyze aDC1 ultrastructural changes related to cell death after incubation with the studied injection solutions. The aDC1 images presented in Figure 3 are representative of one individual and show that before incubation, aDC1s display a typical morphology with intact cytoplasmic and nuclear membranes and the presence of organelles such as mitochondria and endoplasmic reticulum, as well as dendrites (Figure 3(a)). In general, after 4 and 6 hr of incubation with Ringer's lactate and normal saline at 4 and 22°C, it was still possible to observe the presence of organelles, but some cells presented apoptotic characteristics, displaying clear vacuoles or vesicles (Figures 3(b) and 3(c)). However, after 8 hr of incubation, viable aDC1s were still found when incubated with Ringer's lactate at 22°C, whereas with normal saline, it was possible to observe several blebs, showing cellular deterioration (*Supplementary figure 3*). Similar results were achieved for aDC1 incubated at 37°C, in which it was possible to observe modifications that accompany the process of necrosis, including gaps in the cytoplasmic membrane and nuclear fragmentation, with some cells showing loss of contours and structural details after 4 hr of incubation, with any of the solutions studied (Figures 3(b) and 3(c)).

Notably, compared to that of unstored cells, there was a progressive increase in the deterioration of aDC1s maintained in the studied injectable solutions over time, which accelerated when the cells were kept at 37°C.

### 3.4. Maintenance in Normal Saline Disrupts aDC1 Functionality

To evaluate the capacity for secretion of IL-12p70 by aDC1, a proinflammatory cytokine that induces the production of IFN-*γ* by T lymphocytes, promoting differentiation into the Th1 profile, supernatants collected before (unstored cells) and after incubation with normal saline or Ringer's lactate were analyzed by ELISA.

**Figure 4:**
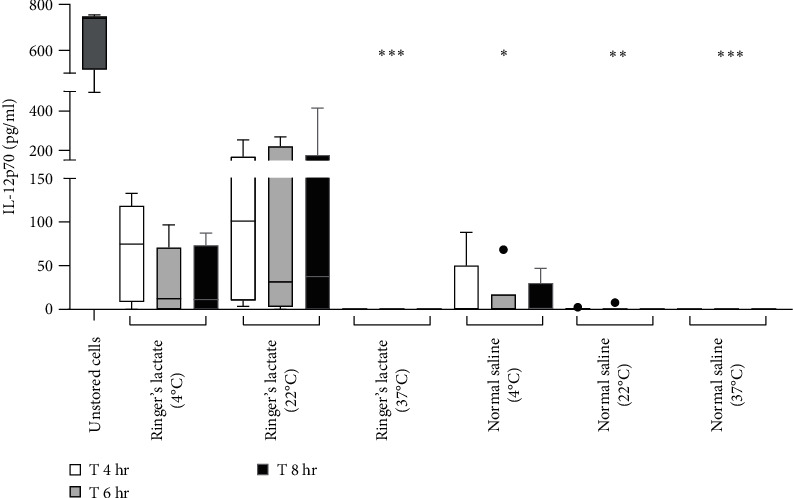
aDC1 production of IL-12p70. Box plots and whiskers (Tukey) representing the concentration in pg/ml of IL-12p70 produced by aDC1s stimulated by CD40L before (unstored cells, hatched bar) and after 4 (T 4 hr) (white bars), 6 (T 6 hr) (gray bars), and 8 hr (T 8 hr) (black bars) of incubation with normal saline or Ringer's lactate at 4, 22, and 37°C (*n* = 6). One-way ANOVA and the nonparametric Kruskal‒Wallis test were used to calculate *p* values.  ^*∗*^*p*  < 0.05;  ^*∗∗*^*p*  < 0.005;  ^*∗∗∗*^*p*  < 0.001.

According to the results presented in Figure 4, before incubation, aDC1 was able to produce an average of 666 pg/ml of IL-12p70. After 4 hr of incubation with Ringer's lactate, the secretion of IL-12p70 by aDC1s reached an average of 67.9 and 101.8 pg/ml when the cells were preserved at 4 and 22°C, respectively. These values decreased to 30.6 and 90.1 pg/ml after 6 hr of incubation, when preserved at 4 and 22°C, respectively, and remained practically unchanged after 8 hr of incubation. On the other hand, compared to those in the initial profile (unstored cells), the IL-12p70 production levels obtained from aDC1s incubated with normal saline were significantly lower after 6 hr at 4°C (11.4 pg/ml) (*p*  < 0.05) and after 4, 6, and 8 hr at 22°C (≤1.3 pg/ml) (*p*  < 0.005). Finally, as expected, due to high cell mortality, no detectable levels of IL-12p70 were detected when aDC1s were maintained at 37°C in normal saline or Ringer's lactate (*p*  < 0.001).

## 4. Discussion

DCs are important targets for immunotherapy treatment, and for several decades, investigators have been developing methods for obtaining and manipulating DCs in experimental protocols for application in a range of clinical settings combined with different treatment agents. Protocols present great variation in various aspects, such as types of DCs and maturation cocktails, sources of antigens, and routes of administration, in addition to the wide genetic diversity and clinical characteristics of patients.

Overall, despite variability, DC immunotherapy elicits some degree of immunological response. In this respect, for all clinical-grade preparations, an aliquot of the final product is obtained prior to injection for quality control assays such as those for viability and immunophenotyping to ensure that cells are alive and activated and for detection of bacteria, fungi, endotoxin, and mycoplasma to guarantee product sterility. Depending on the technical approach, several hours are required to release vaccine product safety results. Since DCs are very responsive to microenvironmental changes, an injectable solution must be adequate to ensure the integrity of DCs during this time.

Normal saline is the most common crystalloid used in fluid therapy, in addition to being extensively used as a solvent for medication delivery [27, 28] and frequently as a vehicle for the injection of therapeutic drugs. In general, for DC-based immunotherapy, most protocols employ normal saline [6, 10, 13, 14, 19, 22, 29, 30, 31, 32, 33, 34, 35] or normal saline containing different concentrations of human serum albumin [3, 4, 7, 12, 36, 37]. Other solutions include PBS containing 5% human serum albumin [8] or 5% autologous serum [38, 39] or autologous serum alone [40]. To standardize a DC production protocol for use in a clinical trial, we tested normal saline as an injectable solution for aDC1, keeping cells at different temperatures for at least 4 hr, which is the average time needed to complete the quality tests.

In addition, considering its ease and practicality and aiming to minimize cell manipulation after quality tests, we also evaluated keeping cells directly in the injection syringe. Despite no change in cell viability, we observed a substantial decrease in aDC1 yield when the cells were stored in syringes, which we speculate can be attributed to the readhesion of cells to the plastic surface of the syringe or loss of cells during aspiration (*Supplementary 2*). In this scenario, considering the difficulty in keeping cells in syringes, the following tests were carried out only while maintaining the cells in ultralow attachment plates.

An interesting aspect addressed here is related to the temperature at which the cells are maintained until inoculation into the patient. Although no differences were detected between cells kept in Ringer's lactate at 4°C or 22°C, cells incubated at 37°C with Ringer's lactate or normal saline showed an increase in death. In this context, often, the final product is kept on ice (2–4°C) [41], but there is little information or guidance available in the literature about this subject. In the present study, when cells were kept at a physiological temperature of 37°C, that is, the temperature at which cells were maintained throughout the differentiation culture, there was a drastic reduction in cell viability that was not observed when these cells were kept at 4 or 22°C. Unlike our findings, even when DCs were maintained for up to 6 hr in saline at 37°C, Marovich et al. [42] did not observe a reduction in DC viability, which was similar to what was observed at 4°C. On the other hand, mesenchymal stem cells maintained in protein-free and nutrient-poor saline presented better viability at low temperatures, which could be explained by the slower cellular metabolism under these conditions compared to that at physiological temperature [43]. Finally, in a study of intracerebral cell implantation, human neural stem cells were maintained for 8 hr under three different conditions: on ice, at room temperature (21°C), and at 37°C; similar to our results, cell viability was maintained only when cells were stored at 21°C [44].

Authors have reported that normal saline is not the most suitable injectable solution for preserving immunotherapy products, despite being widely used as an injectable solution in DC-based immunotherapeutic approaches. Marovich et al. [42] reported that DCs preferred RPMI 1640 medium over other solutions; furthermore, they reported that DC viability was greater when cells were maintained in PBS than when they were maintained in normal saline.

When the pH of normal saline ranges from 4.5 to 7, Ringer's lactate has a less acidic pH (6–7.5) and offers a significantly lower chloride content than normal saline, which may favor the maintenance of cell integrity [45]. In fact, our data showed that Ringer's lactate was able to maintain DC stability at levels similar to those observed under preincubation conditions in terms of viability, maturation status, and IL-12p70 production, and unlike normal saline, no increase in cell death was observed.

Ringer's lactate is a solution used to restore hydration and fluid balance in the body. In this context, a pilot study demonstrated that, compared to normal saline, Ringer's lactate administered for 24 hr significantly reduced the occurrence of systemic inflammatory response syndrome and C-reactive protein levels in patients with acute pancreatitis subjected to fluid resuscitation [46]. These findings were consistent with other clinical trials that similarly identified advantages in administering Ringer's lactate to treat rhabdomyolysis induced by doxylamine intoxication [47] or in the case of renal transplantation [48] in terms of electrolyte balance and pH homeostasis compared with normal saline.

Few groups have used Ringer's lactate in the formulation of vaccine products in the context of DC-based immunotherapy. Cheever and Higano [49] used Ringer's lactate as an injection solution for the first FDA-approved personalized immunotherapy for the treatment of metastatic castration-resistant prostate cancer. This treatment consists of autologous CD54+ cells activated with prostatic acid phosphatase and GM-CSF suspended in Ringer's lactate, and despite inducing a variable-specific response, it has been well tolerated [41, 49].

Another aspect noted in our study is that, in general, the monitoring of the quality of the cells that compose the vaccine product is carried out considering the viability and expression of surface molecules, without taking into account that the apoptosis process may already have been triggered. In the case of maintaining, the cells in normal saline, although those cells had already started the apoptosis process, this could not be visualized in the viability and phenotyping assays. Inoculation of cells already destined for death can compromise cellular performance after inoculation, emphasizing the importance of using an appropriate solution to preserve the viability and quality of the vaccine product.

Apoptosis is a type of nonlytic programed cell death that can be triggered by growth factor deprivation and cellular stress, among many other factors. This process is characterized by morphological changes such as reduced cell size, chromatin condensation, nuclear fragmentation, phosphatidylserine exposure on the cell surface, and apoptotic body formation [50].

Most apoptotic processes involve the activation of a cascade of caspases that mediate upstream signaling events, ultimately leading to the activation of executioner caspases responsible for cellular cleavage [51]. *In vitro* methods employed to investigate apoptosis include, for example, the detection of mitochondrial alterations, the measurement of caspase-3/7 activity, the determination of DNA fragmentation, and membrane damage detection via flow cytometry or microscopic analysis associated or not associated with fluorescent dyes [52]. In the present study, apoptosis was evaluated by flow cytometry after aDC1s remained in Ringer's lactate or normal saline solution for up to 8 hr, with normal saline being responsible for inducing a significant increase in aDC1s during late apoptosis/necrosis, especially when the cells were kept at 37°C. Cellular death induced by a physiological temperature of 37°C was confirmed by TEM, in which cellular morphological changes such as cell condensation, disintegration of the cytoplasmic membrane, and disintegration of the nuclear envelope were clearly observed.

IL-12 is a proinflammatory cytokine that is essential for the interaction between antigen-presenting cells (APCs) and effector cells during the immune response, promoting the differentiation of T lymphocytes into a Th1 profile through the production of IFN-*γ* [53]. Analysis of the secretion of this cytokine by aDC1s before and after incubation with injectable solutions showed that the production of IL-12p70 was achieved only when aDC1s were stored in Ringer's lactate at 4 and 22°C, despite an approximately sevenfold reduction in cytokine levels compared to those in unstored cells.

Several other aspects related to storage and immunization in DC-based immunotherapy that are not addressed here still need to be explored, such as cell density inside the syringe, speed of inoculation in patients, and the best route of administration, which could help in inducing an immune response. Furthermore, the best conditions for keeping the cells inside the syringe, through which the cells must pass to inoculate the patient, remain to be determined. A progressive reduction in cell yield over time may compromise the accuracy of the dose of inoculated cells.

## 5. Conclusion

Taken together, our data showed that, compared with normal saline, Ringer's lactate solution at 4 or 22°C was more effective at maintaining DC integrity and maintaining DC readiness for application. Furthermore, to guarantee the viability and functionality of aDC1s, cells must be inoculated into patients as quickly as possible.

## Data Availability

The authors confirm that the data supporting the findings of this study are available within the article and/or its supplementary materials.

## Supplementary Materials

Supplementary 1 Figure 1: gating strategy for aDC1 analysis. Flow cytometry plot showing the gating strategy for aDC1 analysis.

Supplementary 2 Figure 2: effects of storage on aDC1 characteristics.

Supplementary 3 Figure 3: transmission electron microscopy aDC1 images.
