# Supplementary figures and images for: Effects of Injectable Solutions on the Quality of Monocyte-Derived Dendritic Cells for Immunotherapy
Source: J Immunol Res. 2024 Jun 7;2024:6817965. doi: 10.1155/2024/6817965 (PMC11221978; doi:10.1155/2024/6817965)

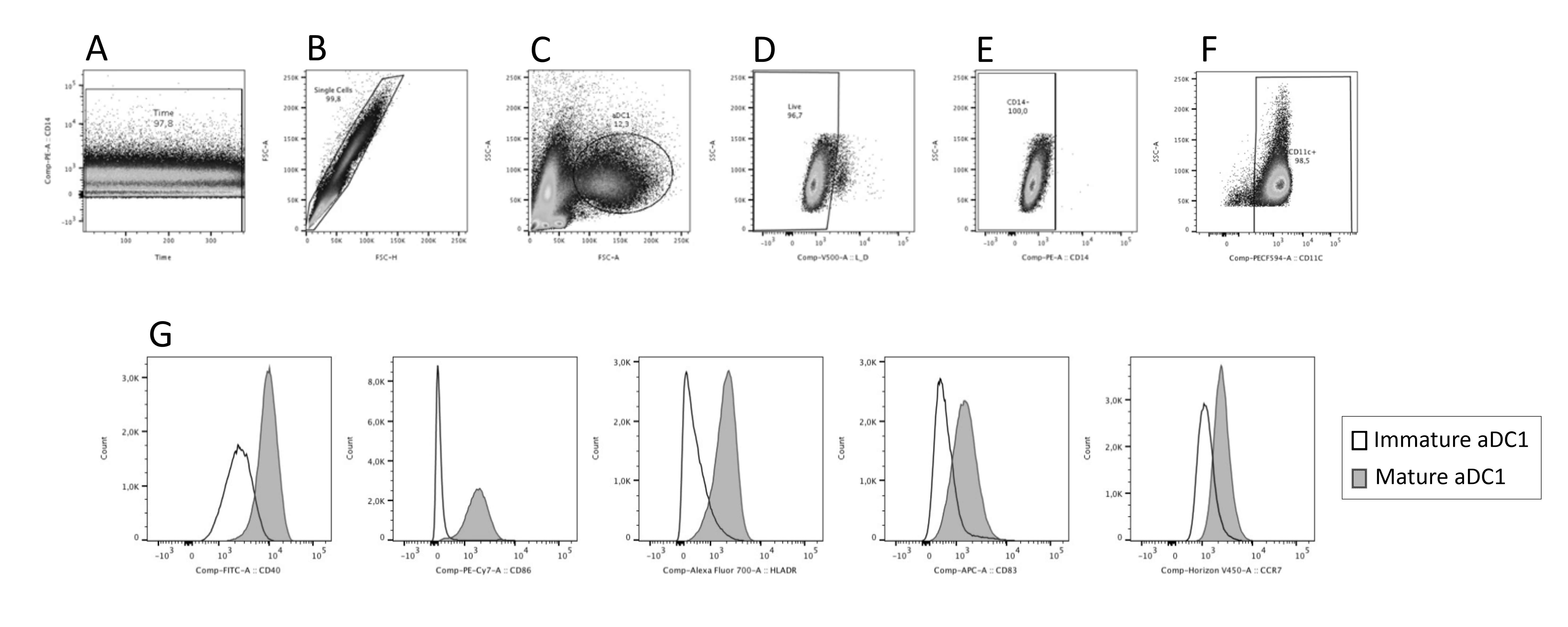

Supplement: Supplementary 1 — Figure 1: gating strategy for aDC1 analysis. Flow cytometry plot showing the gating strategy for aDC1 analysis. [file 6817965.f1.png]

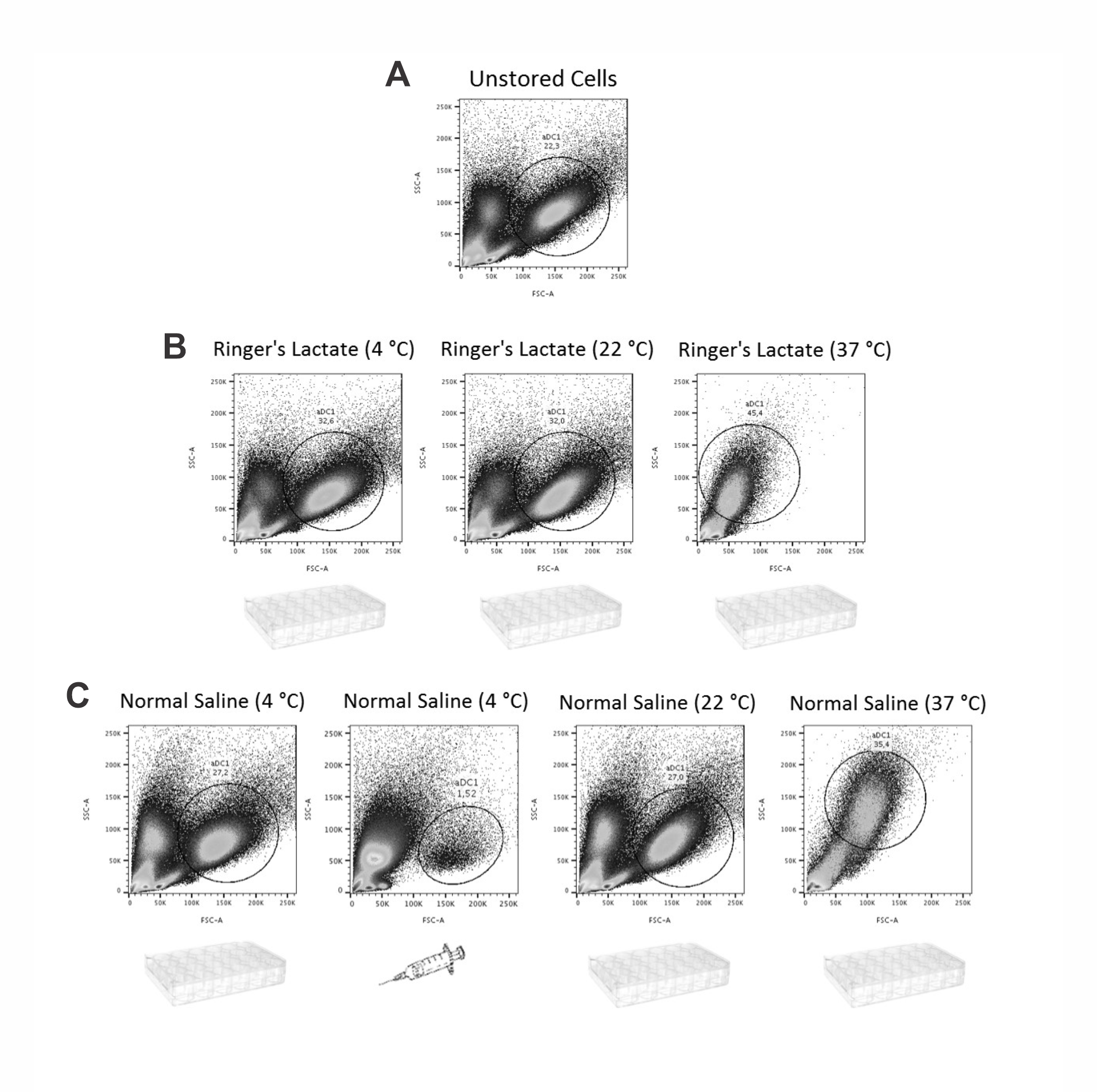

Supplement: Supplementary 2 — Figure 2: effects of storage on aDC1 characteristics. [file 6817965.f2.png]

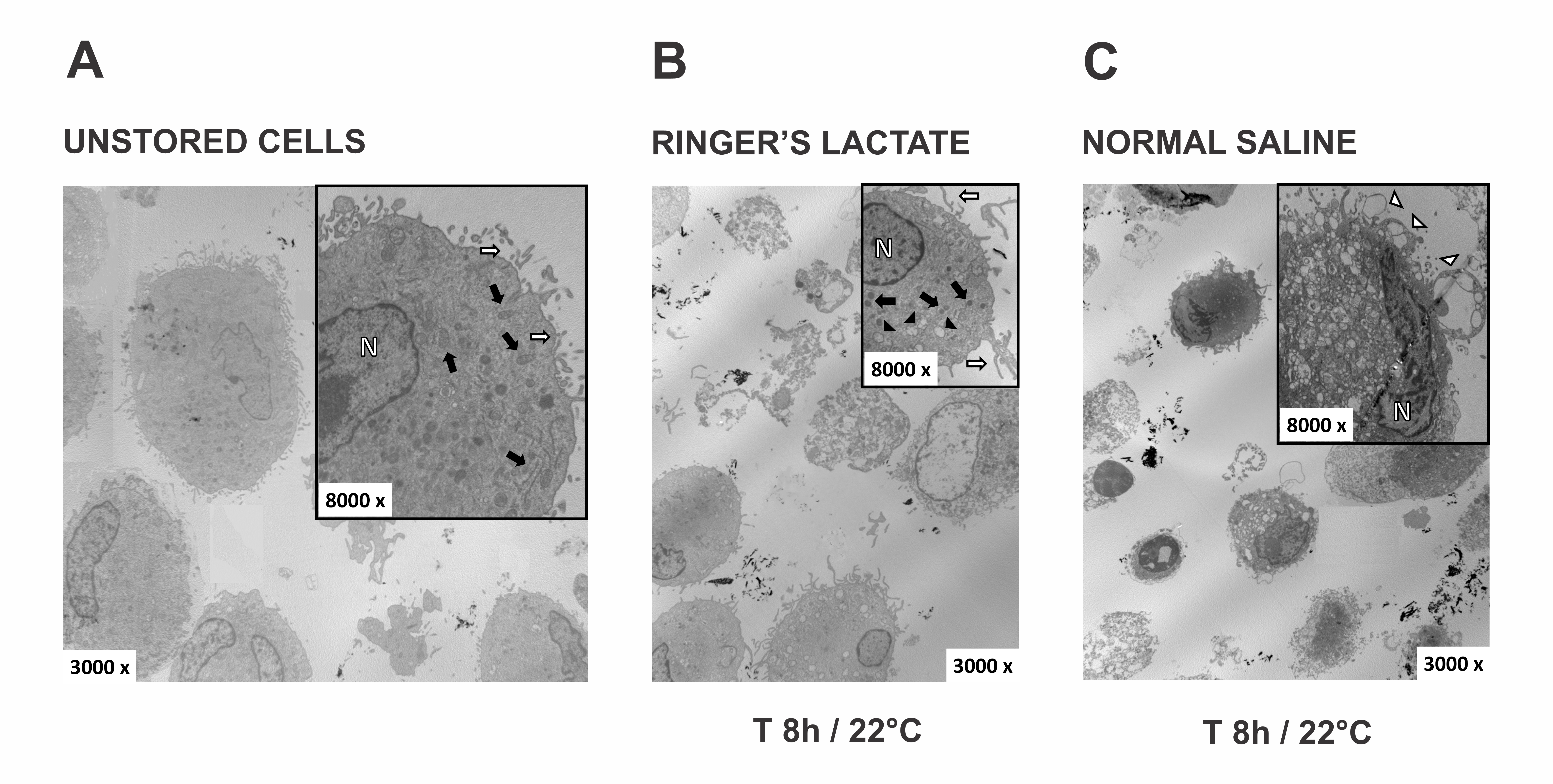

Supplement: Supplementary 3 — Figure 3: transmission electron microscopy aDC1 images. [file 6817965.f3.png]
